# A Biologically-Inspired Symmetric Bidirectional Switch

**DOI:** 10.1371/journal.pone.0169856

**Published:** 2017-01-09

**Authors:** Kahye Song, Shyr-Shea Chang, Marcus Roper, Hyejeong Kim, Sang Joon Lee

**Affiliations:** 1 School of Interdisciplinary Bioscience and Bioengineering, Pohang University of Science and Technology (POSTECH), Pohang, Gyeongbuk, Korea; 2 Department of Mathematics, University of California Los Angeles, Los Angeles, California, United States of America; 3 Department of Biomathematics, University of California Los Angeles, Los Angeles, California, United States of America; 4 Department of Mechanical Engineering, Pohang University of Science and Technology (POSTECH), Pohang, Gyeongbuk, Korea; University of Akron, UNITED STATES

## Abstract

Stimuli-sensitive hydrogels have been intensively studied because of their potential applications in drug delivery, cell culture, and actuator design. Although hydrogels with directed unidirectional response, i.e. capable of bending actuated by different chemical components reaction in response to several stimuli including water and electric fields, these hydrogels are capable of being actuated in one direction only by the stimulus. By contrast the challenge of building a device that is capable of responding to the same cue (in this case a temperature gradient) to bend in either direction remains unmet. Here, inspired by the structure of pine cone scales, we design a temperature-sensitive hydrogel with bending directed an imposed fishing line. The layers with same PNIPAAm always shrinks in response to the heat. Even the layers made with different chemical property, bends away from a warm surface, whether the warm surface is applied at its upper or lower boundary. To design the bending hydrogel we exploited the coupled responses of the hydrogel; a fishing line intercalating structure and change its construction. In addition to revealing a new capability of stimulus sensitive hydrogels, our study gives insight into the structural features of pine cone bending.

## Introduction

Stimulus-responsive can change their shapes, elastic and surface properties, as well as chemical characteristics like solubility in response to external stimuli, and they have found applications in drug delivery and cell culture, as well as sensors, pumps and actuators[1–5]. One of the biggest challenges in stimuli-responsive materials is motion control for smart functional materials and actuators[6–11]. In many applications hydrogel-materials have been designed that are capable of moving in a pre-determined direction when subject to external cue; for example a strips that fold when wetted and then unfold during drying, these kinds of devices may be used as switches, cantilevers or for flow control in micro-fluidic devices[6, 12]. However, the anisotropic motion has been achieved though the anisotropy of the structure as well as solvent gradient: they are capable of bending only in one direction in response to the cue that they receive: the archetype of such a device is a bimetallic switch, composed of two materials with different coefficients of thermal expansivity, that bends in one direction when heated. An alternate possibility, less discussed, is that a device that receives a weakly anisotropic cue and is capable of altering its shape in different directions depending on the direction of this gradient. The structure of this device would need to be symmetric, but capable of responding to potentially small gradients of stimulus.

**Fig 1:**
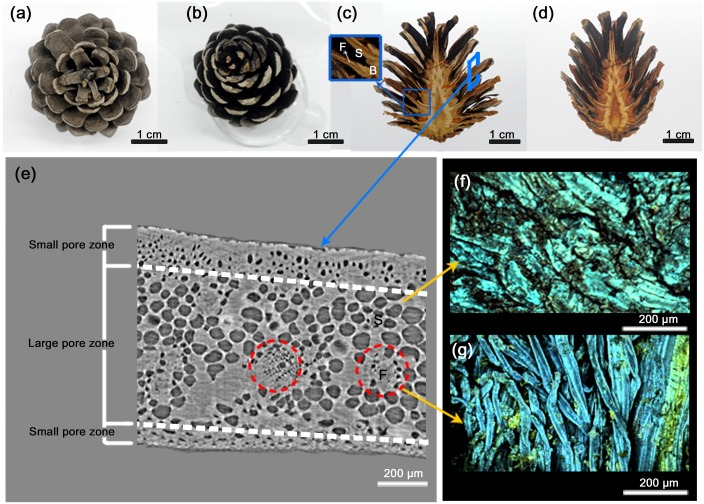
Morphological characteristics of pine cone scales. **(a)** Pine cone scales fully open on sunny dry days. **(b)** When pine cone gets wet, pine cone folds its scales **(c)** Hemisected pine cone shows layered structure. In dry condition, scales are fully opened. Pine cone scale consists of fibers (F) and sclerids (S). ‘B’ signifies bract scale. **(d)** Pine cone fold its scale after wetting. **(e)** Cross sectional image of scale is captured using X-ray microscopy. The large pores are surrounded by small pores like layer structure. Fibers penetrate into the scale structure. ‘S’ and ‘F’ signify sclerids and fibers, respectively. Anatomic 3D structure of sclerid **(f)** and fiber **(g)** are investigated 3-dimensionally using multi-photon microscopy. The sclerid has dense structure like rock, while the fibers are tangled up.

The nature has already shown us a potential solution: the bending of the pine cone scale. Seed-bearing pine cones open in sunny dry weather and close in humid condition (Fig 1a–1d)[13]. Since pine cone consists of dead cells, its dynamic motion is cause by morphological changes of scales. Although usually modeled as bimetallic structures (that is, made up of two mechanically different layers), pine cone scales are in fact composed to three different layers of dead cells, with an inner layer containing large scale pores, and stiffened by aligned cellulose fibrils sandwiched between two layers that contain small-scale pores (Fig 1e–1g)[14, 15].

We fabricated a THIP (Temperature gradient responsive Hydrogel Inspired by Pine cone) PNIPAAm mimic of the pine cone scale that is capable of bending in response to temperature gradients. This study brings us one step closer to understanding the pine cone motile mechanism and can be applied in various industrial fields as symmetric materials but capable of responding to potentially small gradients of stimulus.

## Methods

### PNIPAAm synthesis

Photocrosslinkable PNIPAAm was synthesized using the free-radical polymerization method. 100mg of N-isopropylacrylmide monomer (NIPAAm) (Sigma Aldrich, USA) was dissolved in 0.6 ml of deionized water to preparing a pre-gel solution. In this monomer solution, 1mg of 2-hydroxy-1-[4-(hydroxyethoxy)phenyl]-2methyl-1-propanone (Sigma Aldrich, Iragacure 2959, USA) was added as a photoinitiator. In this study, two different concentrations of crosslinker (C1 and C3) were use: 1 mg and 3 mg of *N*,*N’*-methylenebisacrylamide (MBAm) (Sigma Aldrich, USA) were dissolved in 0.1 ml of deionized water.

The 700 μl pre-gel solution was poured into a Polydimethylsiloxane (PDMS) (Dow Corning Corp., USA) mould. Then, 1mm supporting beams were installed along the PDMS mould wall for height equalization. The solution covered with a film was irradiated for 8 min with 365nm UV light (VIRVER Lourmat-4.L, France). In the illuminated regions, free radicals leads to polymerization.

### Source of pine cone

The tested pine cones were from Japanese red pine (Pinus densiflora), which is common in Korea, Japan and northeastern China. The pine cones were collected from POSTECH campus (Pohang in Korea) in spring. Pine cones were cut in half using a wire saw and captured using a digital camera (Nikon D700, Japan).

### X-ray tomography

Cross sectional images of the pine cone scale were observed using X-ray micro computed tomography at the 6C beamline of the Pohang Accelerator Laboratory without any chemical treatment. To minimize the photo thermal damage to the test sample, a 23 mm filter and a 1.5 mm-thick silicon wafer was installed in the pathway of X-ray beam pathway. The distance from the test sample to the camera was set to 20 cm. The X-ray images were consecutively acquired using a sCMOS camera with 2560 × 2160 pixels (Andor Zyla, UK). Based on the pixel size of the camera with a ×4 objective lens, the spatial resolution was evaluated 1.6 μm/pixel and the field of view (FOV) was 4 mm × 3.5 mm. The test sample was fixed to a sample holder when the stage was rotated from 0° to 180° at 0.5° intervals.

A tomogram of each acquired image made to sinogram using the modified Bronnikov algorithm (MBA) filter of the Octopus software (inCT, Belgium) to remove erroneous spots in the captured X-ray images and reconstruct with better a spatial resolution.

### Image acquisition using confocal laser scanning microscopy

Sclerids and fibers of the pine cones were observed using a confocal laser scanning microscopy (Leica Microsystems Ltd. TCS SP5 II MP with SMD, Germany) with a HC PL FLUOTAR 10× objective lens (Leica Microsystems Ltd. HC PL FLUOTAR 10×0.3 DRY, Germany) without any chemical treatment. The samples were magnified using a 20× zoomed lens and the corresponding FOV was 775 μm × 775 μm × 408 μm. By using the 3.4 kW (780 nm) laser power, the structures were consecutively captured with 3 μm depth interval and the total exposure time was 185 seconds.

The acquired images were analyzed and processed using the LAS AF 2.7 software (Leica Microsystems Ltd. Germany). Outlier noises were removed by using filters of Image J software (National Institutes of Health, USA) to improve the image quality. The consecutively captured images were reconstructed into a 3D morphological structure.

### Synthesis of triple PNIPAAm layers embedded with fishing lines

The 700 μl pre-gel solution was poured on the fabricated PNIPAAm single layer. To make the layers embedded with fishing lines (FE), 3 fishing lines (polyamide 6, Fastline, Korea) of 10 mm in diameter were positioned in the middle of the pre-gel solution; for effective bending motion, a fishing line of 10 mm diameter is required for the layers of 5 mm × 7.5 mm. Then, 1mm supporting beams installed along the PDMS mould wall. The 365nm UV light was used for irradiation over the film covered pre-gel solution for 8 min. After hardening, another 700 μl pre-gel solution was poured on the hardened double layer and repeated a series of fabrication process. Lastly, fabricated fishing line embedded triple layers were carefully separated from PDMS mould and cut into 15 mm × 7.5 mm in physical dimension.

### Heating PNIPAAm and data acquisition

After fabrication, PNIPAAm layers were fully swollen with deionized water. Since, the PNIPAAm layer is transformed over 32℃, fully hydrated PNIPAAm layers were heated at 36℃ using a Peltier chip. The transformation process of PNIPAAm was captured consecutively using a digital camera (Nikon D700, Japan) at time intervals of 10 seconds for 30 minutes. Acquired data was quantitatively analyzed using Image J software (National Institutes of Health, USA) to evaluate the breadth, length and surface area.

### Tensile strength experiment for Young’s modulus evaluation

Variation of Young’s modulus before/after heating the single PNIPAAm layer was evaluated using a universal testing machine (Instron 3344, USA). The three dimensional sizes of test samples were measured before and after the tensile experiment for calculating Young’s modulus. The lower part of the sample was fixed with clampers and pulled upward with 5 mm/min velocity. Young’s modulus was calculated using the following formula,

$$E=\frac{\sigma }{\varepsilon }=\frac{F_{l}/A}{\Delta L/L}$$

Where *E*, *σ* and *ε* denote Young’s modulus, stress and strain value, respectively. *F*_*l*_ is the force measured by a load cell and *A* is the tension received surface area. *L* signifies initial length of the sample and *ΔL* is the extended length.

## Results

### Constitutive response of PNIPAAm hydrogels to heating

**Fig 2:**
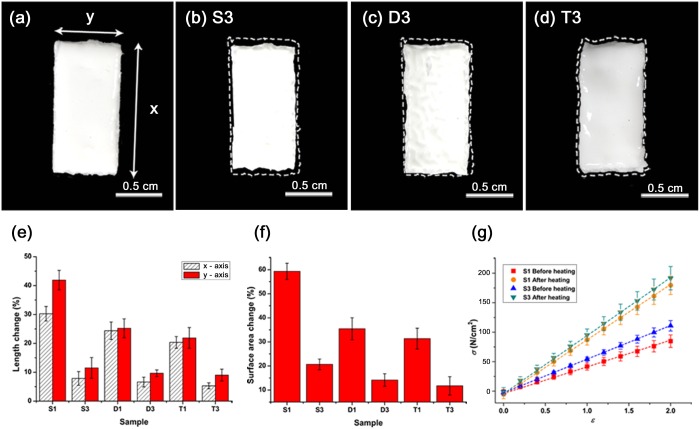
Homogeneous PNIPAAm structures shrink in response to heat. **(a)** The homogeneous PNIPAAm structures have the same size at the beginning. The fabricated PNIPAAm structures with 3 mg cross linker as single **(b)**, double **(c)** and triple **(d)** layers are shown. After heating, single **(b)**, double **(c)** and triple **(d)** layers shrink in every direction. White dash lines indicate the initial size of each structure. The fabricated structures do not bend, but shrink. **(e)** Length changes in length in the x- and y-directions for different PNIPAAm structures were depicted as a graph. S1 (single layer with C1 cross linker. Other structures are named with the same rule.) shows the most significant structural change and the T3 exhibits least change. **(f)** Surface area changes of S1, S3, D1, D3, T1 and T3 were measured and depicted as a graph. Like length variation, S1 shows the biggest change and T3 shows the least change. **(g)** Young’s moduli of S1 and S3 change in response to heating depicted as graph. The Young’s modulus of dense S3 is higher than that of S1. Since heating increases the stiffness of PNIPAAm, the Young’s modulus also increases.

Poly(N-isopropylacrylamide) (PNIPAAm) can be used to create hydrogels that change their size in response to multiple stimuli. In particular, when it is heated, PNIPAAm loses water and shrinks. This process is reversible: provided water is present, PNIPAAm will reabsorb water on cooling, and return to its original size. At the same time, alterations occurring to the elastic matrix of the hydrogel cause the mechanical properties of the hydrogel to change when it is heated. We characterized the response of homogeneous PNIPAAm layers to temperature gradients, by placing layers of PNIPAAm on a heated Peltier chip maintained at 36℃. We varied both the thickness of each strip (in this work a single thickness, 0.5 mm, strip refers to a single PNIPAAm layer), and the amount of crosslinker added to the gel (throughout this study we used two different concentrations: 1 mg and 3 mg in 700 μl of gel solution, which we refers to as C1 and C3 strips respectively) (Fig 2a–2d): because of different amount of crosslinker, the layers were fabricated with different degrees of polymerization[5, 16]; larger amounts of crosslinker tend to produce a more tightly woven gel mesh.

When the PNIPAAm layers are heated, they release water and shrink by a significant fraction (10–40%) of their initial length in every direction (Fig 2e). Large-meshed single strips created with C1 (S1) shrunk approximately 3 times more than single strips created with C3 (S3) (Fig 2f).

Heating also increased the stiffness of PNIPAAm layers. We measured the Young’s modulus of single layer strips before and after their heating using a universal testing machine (Instron 3344, Instron Ltd., Norwood, MA, USA) (Fig 2g) Strip stress was measured under a prescribed extension. We found that heated PNIPAAm layers increased their Young’s modulus by a factor of approximately two, but that there was slight difference in Young’s modulus between C1 and C3 strips, whether they were heated or unheated.

Interesting, thicker strips (double layer with C1 (D1), triple layer with C1 (T1), double layer with C3 (D3) and triple layer with C3 (T3)) shrunk, less on average, than thinner strips (S1 and S3). We compared the amount of shrinkage between single, double and triple layers of homogeneous PNIPAAm gel, using either C1 or C3 for all layers. All strips shrunk in response to heating, but increasing the strip thickness decreased the rate at which shrinking occurred, as well as the asymptotic (maximal) amount of shrinking (Fig 2e and 2f).

A temperature gradient in the z-direction will cause different z-surfaces to shrink by different amounts. If the difference between shrinkage on heated and cooled surfaces is large enough, then strips may potentially bend. However, the amount of bending is too small to be experimentally measured—in particular, we saw no evidence of any part of the strip lifting off the Peltier chip. One widely studied principle for converting shrinkage to bending, is to bond two different layers of hydrogel together, with different coefficients of thermal shrinkage. A two-layer hydrogel strip will show strong differentials in shrinkage, even when both material layers are maintained at the same temperature [3, 6, 11]. Then, like a heated bimetallic strip, the two-layer strip will bend, with the less shrunk layer (smaller shrinkage coefficient) forming the outside of the curve, and the more shrunk layer (larger shrinkage coefficient) forming the inside of the curve. Indeed, two layer strips containing one layer of C1, and one containing C3, scrolled up rapidly in response to heating. Although two-layer hydrogels have been used as models for biological stimulus responsive levers and ratchets[17], we would expect such a hydrogel to bend in the same direction, independently of the temperature gradient; that is the direction of bending is determined by the mismatch in shrinkage coefficients. For a symmetric strip to bend, we expect that is should be needed secret structure, but it is not clear how bending can occur in the presence of this symmetry.

### Mimicking the pine cone scale to create consistent bending

To create bending, we exploit both aspects of the response of the PNIPAAm layers to heating: (1) shrinkage and (2) a change in elastic modulus. We designed hydrogel structures made up of three layers: two layers of 3 mg cross-linker, intercalated by a single layer containing C1 (Fp). When homogeneously heated, the middle layer will shrink more than the surrounding two layers. This alone does not lead to bending, because strains are symmetric in all three layers; we therefore looked to a biological hydrogel—the pine cone scale—as an example of symmetric multi-layer stimulus sensitive material that is capable of bending. On sunny dry days, the pine cone scales open, allowing seeds to be dispersed (Fig 1a and 1c)[18], on wet days, the scales close again (Fig 1b and 1d) with symmetric structure.

**Fig 3:**
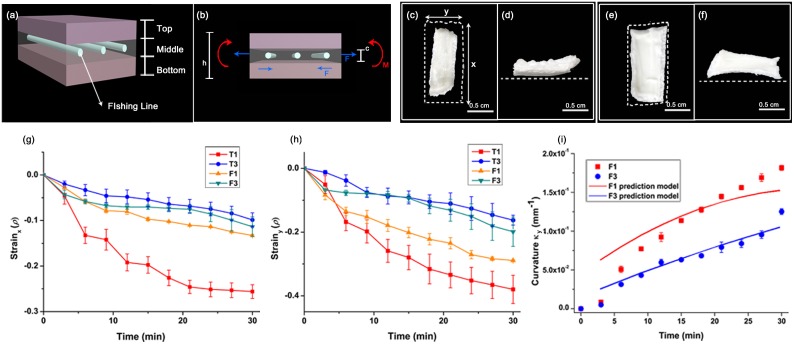
Homogeneous PNIPAAm with fibers show significant bending. **(a)** Schematic diagram of the proposed PNIPAAm structure inspired by pine cone scale. Fishing lines are embedded in the middle layer representing fibers in the pine cone scales. **(b)** Schematic diagram for a bimetallic theory considering the fiber constraint and the temperature gradient. In the diagram, *h* is the height of the system, *c* is the thickness of the part constrained by fishing lines, and *F*_*1*_, *F*_*2*_ and *M* are the internal forces and torque caused by the temperature gradient. The middle and the bottom parts are in higher temperature than the top part. **(c-f)** Structural changes caused by heating of F1 (triple layer with fishing lines and C1) and F3 (triple layer with fishing lines and 3 mg cross linker). (**c**) White dash lines indicate the initial size of the structure. After heating, F1 mainly shrinks in y-direction. (**d**) Instead of shrinkage in x-direction, the side view shows upward lifting motion of F1 layers. (**e**) F3 structure slightly shrinks from its initial size (white dash lines). (**f**) F3 exhibits significant bending motion, like F1 structure. Strain changes in the x-direction (parallel to fiber direction) **(g)** and in the y-direction (perpendicular to fiber direction and the temperature gradient direction) **(h)** depicted as graphs. Due to the support of the fishing line in the x-direction, the strain change with temperature of F1 is much less than that of T3. **(i)** The curvature in the direction parallel to the fibers can be qualitatively explained by a bimetallic theory combining the fiber constraint and the temperature gradient. Dot: experimental measured curvature of PNIPAAm layer with fishing lines (square: F1, circle: F3) in y-direction over time. Line: theoretical prediction with linearized contraction measurement on PNIPAAm layers without fishing lines (T1, T3). The thickness of the fiber-constrained layer *c* fitted from the curvature data are 0.9 mm for F1 and 0.67 mm for F3, compared to the height 1.5 mm of both strips.

Although they have previously been modeled as two layer structures[15], pine cone scales are actually symmetric, being made up of a sandwich of three layers with different pore sizes, and that shrink differently under desiccation (Fig 1e). The important key structure is in middle layer, containing the largest pores is run through with stiff fibers (Fig 1e). We built PNIPAAm structures to mimic both features of the real pine cone scale: (1) a sandwich structure with the largest pore material in the middle of the sandwich (2) stiffening fibers that prevent extension of middle layer in one direction. To introduce stiffening fibers into our strips, we created PNIPAAm layers embedded with parallel nylon fishing lines (Fig 3a), and built sandwich structures, in which all three layers contained the same amount of cross-linker, and the inner layer also contained parallel fishing lines(C1 with fishing line contained triple layer (F1) and C3 with fishing line contained triple layer (F3). Before heating, the shapes of these tri-layers were the same as homogeneous tri-layers (T1, T3 (that is, not containing fishing line)), but as anticipated, both F1 and F3 strips showed anisotropic stretching; shrinking by a much smaller factor than the corresponding homogenous strips in the direction of the fishing lines and by a much larger factor in a direction perpendicular to the fishing lines (Fig 3c–3f). We interpret the increased shrinkage of strips in the direction perpendicular to the fishing lines to result from compensation of strips for their inability to shrink in the direction of fibers, increasing the amount that they shrink in directions that are not constrained by the fibers. Importantly, unlike both homogenous and heterogeneous layers (that is strips in which both all layers contained the same amount of cross-linker, and layers in which the C1 strip was sandwiched between C3 strips), incorporating fishing line in the inner layer caused it to bend as well as shrinking in response to heating (Fig 3c–3h). In addition, the amount of fishing lines decisively contributes to the layer’s bending motion. When the amount of fishing lines embedded to the layers in doubled, the layers do not bend or shrink, while keeping the shape. When the layers reach the limit, they are torn and the fishing lines are popped up. Since a large amount of fishing lines makes the layers stiff, the layers lose the flexibility for bending. On the other hand, without embedding fishing lines, the layers shrink in every direction and do not bend at all. For inducing bending motion, the embedment of a particular amount of fishing lines is required.

Why does the PNIPAAm sandwiches in which the middle layer contains fibers show qualitatively different motion compared with triple layers without fiber stiffening? We align the x-axis with the direction of fibers, the y-axis perpendicular to the fibers, but in a plane parallel to the heating surface, and the z-axis with the depth dimension (perpendicular to the heating surfaced). Consider the yz-cross section of a PNIPAAm layer that contains fibers (Fig 3b). Since fibers prevent shrinkage in the x-direction, this middle layer shrinks more than the surrounding layers in the y-direction (Fig 3g and 3h). We may then adapt the theory of bimetallic strips to analyze the bending that heterogeneous strains can produce. Suppose that the height of the strip is *h* and the thickness of the middle layer constrained by the fibers is *c*, which would be determined by the experimental data in different structures. We may then use the shrinkage factors measured for homogenous strips as well as the already noted temperature dependence of stiffness (Fig 2g). The middle layer shrinks more than either upper or lower layers, so if only the middle and lower layers were present then the strip would bend downward, whereas if only the middle and upper layers were present then we would expect it to bend upwards, in both cases analogous to the bimetallic strip, with the middle layer acting as the shorter side of the strip. In the case where both upper and lower layers are present we would expect no bending to occur, if both the upper and lower layers had the same stiffness. However, heating increases the stiffness of PNIPAAm: since under our temperature gradient model, the lower layer has higher Young’s modulus than the upper layer. Since the upper layer therefore does less to oppose the extension of the middle layer, the bottom layer becomes the long side of the bimetallic strip, and neglecting the contribution of the most extensible upper layer. We can calculate, quantitatively, the curvature by adapting the theory of bending of bimetallic strips[15, 19]. Specifically on the boundary between the middle and the bottom layers the strain has to be the same either calculated from the middle or the bottom layer, which implies

$$\alpha_{1}-\frac{2F_{1}}{E_{1}(h-c)}-\frac{h-c}{4\rho }=\alpha_{2}+\frac{F_{1}+F_{2}}{E_{1}c}+\frac{c}{2\rho }$$ (1)

Where *α*_*1*_ and *α*_*2*_ are the strain of the bottom, middle parts respectively, *E*_*1*_ is the planar Young’s modulus of the heated parts, *F*_*1*_ and *F*_*2*_ are respectively the force on the bottom and top layers resulting due to different layers being constrained from shrinking to their respective natural lengths from imbalance of contraction between the middle and the bottom parts, *h* is the height of the whole layer, and *ρ* is the radius of curvature (Fig 3b). Similarly, on the boundary between the middle and the top layers we have

$$\alpha_{2}+\frac{F_{1}+F_{2}}{E_{1}c}-\frac{c}{2\rho }=\alpha_{3}+\frac{2F_{2}}{E_{2}(h-c)}+\frac{h-c}{4\rho }$$ (2)

Where *E*_*2*_ is the planar Young’s modulus of that parts in the ambient temperature, and *α*_*3*_ is the strain of the top part. The forces *F*_*1*_ and *F*_*2*_ would have to balance with the bending moment *M* of the strip, which leads to

$$\frac{h+c}{4}(F_{1}-F_{2})=\frac{E_{1}I_{1}+E_{1}I_{2}+E_{2}I_{1}}{\rho }$$ (3)

where

$$I_{1}={\left(\frac{h-c}{2}\right)}^{3}\frac{1}{12},\;I_{2}=\frac{c^{3}}{12}$$ (4)

The *E*_*1*_*I*_*1*_, *E*_*1*_*I*_*2*_ and *E*_*2*_*I*_*1*_ represent the flexural rigidity of the bottom, middle and the top parts. We can solve for *F*_*1*_ and *F*_*2*_ by Eqs (1 and 2):

$$F_{1}=\frac{1}{\Delta }\left\{\frac{1}{E_{1}c}(\alpha_{1}-\alpha_{3}-\frac{h+c}{2\rho })+\frac{2}{E_{2}(h-c)}(\alpha_{1}-\alpha_{2}-\frac{h+c}{4\rho })\right\}$$ (5)

$$F_{2}=\frac{1}{\Delta }\left\{\frac{1}{E_{1}c}(\alpha_{3}-\alpha_{1}+\frac{h+c}{2\rho })+\frac{2}{E_{1}(h-c)}(\alpha_{3}-\alpha_{2}+\frac{h+c}{4\rho })\right\}$$ (6)

Where

$$\Delta =\frac{2}{E_{1}E_{2}c(h-c)}+\frac{2}{E_{1}^{2}c(h-c)}+\frac{4}{E_{1}E_{2}{\left(h-c\right)}^{2}}$$ (7)

Plugging Eqs (5 and 7) into Eq (3) could give us the curvature $\kappa =\frac{1}{\rho }$. To emphasize the effect of the difference in the Young’s modulus we could consider the limit *E*_*2*_ → 0. Under this limit we would have

$$\frac{h+c}{4}(F_{1}-F_{2})\to \frac{c(h-c)E_{1}}{4}(\alpha_{1}-\alpha_{2}-\frac{h+c}{4\rho })$$ (8)

$$\frac{E_{1}I_{1}+E_{1}I_{2}+E_{2}I_{1}}{\rho }\to \frac{E_{1}(I_{1}+I_{2})}{\rho }$$ (9)

Then we can obtain the curvature by plugging Eqs (8 and 9) into Eq (3):

$$\kappa =\frac{1}{\rho }=\frac{\frac{c(h-c)}{4}(\alpha_{1}-\alpha_{2})}{\frac{c(h^{2}-c^{2})}{16}+\frac{1}{12}{\left(\frac{h-c}{2}\right)}^{3}+\frac{c^{3}}{12}}$$ (10)

Bending upward corresponds to positive *κ*, which happens when *α*_*1*_ > *α*_*2*_. In our notation *α*_*1*_, *α*_*2*_ are both negative indicating both layers are contracting, and larger absolute value means greater contraction. Since the middle part is constrained in x-direction it would contract more in the y-direction, so the layer would always bend to the colder side, in agreement with the experiments. If we denote *α*_*x*_, *α*_*y*_ to be the unit strain in the x, y-directions, and *α’*_*y*_ to be the unit strain when constrained in x-direction, then assuming that the contracted areas are the same for fiber stiffened as well as for non-stiffened strips then:

$$\alpha_{x}+\alpha_{y}+\alpha_{x}\alpha_{y}=\alpha \prime_{y}$$ (11)

Eqs (10) and (11) relate the curvature of the sandwich layer to the strains measured for PNIPAAm that do not have fiber stiffening the contraction measurements of that without fishing lines in x, y-directions, and the thickness of the middle part *c* is determined by fitting to the data. The theoretical prediction qualitatively agrees with the experimental measurement (Fig 3i). Although details of the model could be modified to improve the prediction, a more important aspect of this model is that it captures the interaction between the temperature gradient and the fiber constraint, and explains how the temperature-directed response of a homogeneous PNIPAAm layer is possible.

**Fig 4:**
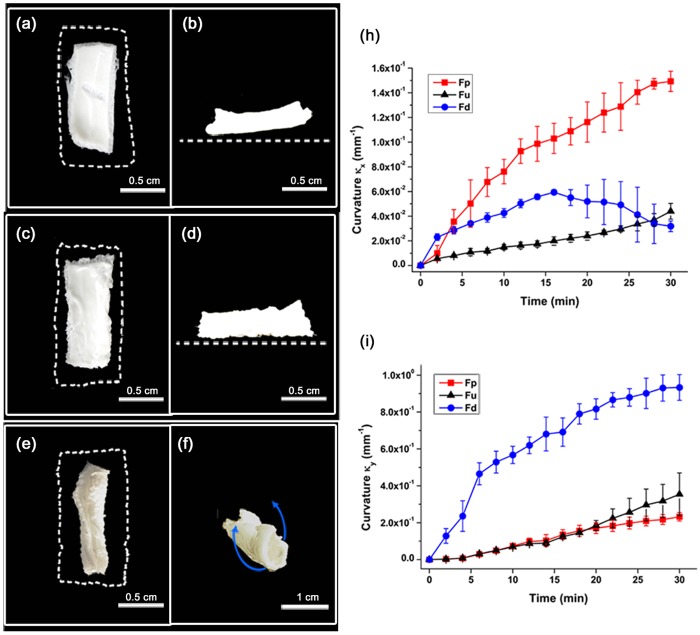
Motion of various layered structures (a-b). The pine cone inspired model (Fp) lifts up its body after heating. **(a)** The structural change of Fp is observed in the top view and white dash lines represents the initial size of the structure. **(b)** The lifting height is clearly shown in the side view. **(c)** Heterogeneous (C1-C3-C1) triple layers (Fu) becomes crinkly in response to heating. **(d)** One side is slightly lifted. **(e-f)** After heating, the topless structure (Fd) rolls up. Thus, the structure is capable of bending in both directions. **(h-i)** Temporal variations of the curvature in the x-direction and y-directions during heating. **(h)** The Fp structure bends the most in the x-direction compared to other structures. **(i)** The Fd structure bends the most in the y-direction. However its asymmetric structure would not allow it to bend according to the direction of the temperature gradient, which Fp and Fu structures are capable of.

Our mathematical model suggests that the strongest bending occurs when the middle layer of the strip layer has the largest possible contraction. Within the pine cone scale, the outer-most scales have smaller pores (approximately 50 μm) than the middle layer (whose pores are approximately 150 μm) (Fig 1e)[14]. Our PNIPAAm layers bent consistently due to the mismatch of strains between middle and lower layers, so we reasoned that greater bending could be achieved if, like the pine cone, the middle layer has a larger pore size than the outer layers, thereby increasing the mismatch between the strains in the two layers (Fp). In PNIPAAm layers this pore size is controlled by the amount of cross-linker. Accordingly, we built heterogeneous (C3 –C1 –C3) triple layers (Fp) in which the fiber-stiffened middle layer contained C1 of cross-linker (producing large pores) and the two outer layers contained C3 of cross-linker (producing small pores).The bending motion of these strips exceeded bending in strips in which a fiber stiffened middle layer was sandwiched between two layers that had the same pore size (Fig 4a and 4b). As a control, we also created heterogeneous (C1 –C3 –C1) triple layers (Fu), in which the ordering of pore sizes was reversed (Fig 4c and 4d). These strips bent significantly less than the strip mimicking the pinecone scale (Fig 4h). However, all strips (heterogeneous and homogeneous triple layers) bent significantly less than strips in which the upper layer was removed (Fig 4e, 4f and 4i) (Fd). This comparison is not surprising, since the unstrained contraction of the upper layer is similar to that of the lower layer, it opposes the bending of the strip, but it underscores the point that a symmetric-temperature gradient responsive strip, that is capable of bending in both directions, bends less than an asymmetric strip that is designed to bend in a single direction only (Fig 4h and 4i).

## Discussion

In this study, we designed a temperature-sensitive hydrogel with symmetric structure with inspiration from pine cone scales. Importantly, owing to fishing line, this artificial symmetric pine cone scale always bends away from a warm surface, is capable of bending in either direction and bends more than an order of magnitude more than a single layer material is capable of doing. The developed system can be applied to various fields, because the direction and strain of the system can be easily controlled.

Within the biomimetic pine cone scale, the bending motion is caused by the combination of fiber constraint and the temperature gradient. Surprisingly the PNIPAAm layer with fibers does not bend toward the side with higher temperature, as we would expect if differential shrinkage caused by temperature gradients alone were responsible for the bending. Indeed we found that homogenous strips bent negligibly. And you can use this structure with any other chemicals which are response to temperature, humidity and so on.

In addition to designing a symmetric temperature-sensitive hydrogel, our study also suggests that the array of fibers and the triple layered structure might play an important role in the bending motion of pine cone scales. A direct experimental investigation of the structural effect on pine cone scale bending is difficult due to the hardness of pine cone scale. Our method allows ready construction of hydrogels with different structures to measure the bending efficiency, and we can verify the theoretical predictions against the empirical data. We expect our study to not only be a bio-inspired system but serve as an attempt to unravel the underlying principles in the design of pine cone scales.
