# Correction: A Biologically-Inspired Symmetric Bidirectional Switch

**DOI:** 10.1371/journal.pone.0204700

**Published:** 2018-09-20

**Authors:** Kahye Song, Shyr-Shea Chang, Marcus Roper, Hyejeong Kim, Sang Joon Lee

The affiliation for the fifth author is incorrect. Sang Joon Lee is not affiliated with #1 and #3 but with #1 School of Interdisciplinary Bioscience and Bioengineering, Pohang University of Science and Technology (POSTECH), Pohang, Gyeongbuk, Korea, and #4 Department of Mechanical Engineering, Pohang University of Science and Technology (POSTECH), Pohang, Gyeongbuk, Korea.
